# Predictors of Bone Mineral Density among Asian Indians in Northern Mississippi: A Pilot Study

**Published:** 2016-11-05

**Authors:** Vinayak K Nahar, Kyle M Nelson, M Allison Ford, Manoj Sharma, Martha A Bass, Mary A Haskins, John Garner

**Affiliations:** ^a^ Department of Health, Physical Education, and Exercise Science, School of Allied Health Sciences, Lincoln Memorial University, Harrogate, TN, USA; ^b^ Bone Mineral Density Laboratory, Department of Health, Exercise Science & Recreation Management, School of Applied Sciences, University of Mississippi, MS, USA; ^c^ Behavioral & Environmental Health, School of Public Health, Jackson State University, MS, USA; ^d^ Department of Kinesiology and Health Promotion, College of Health & Human Services, Troy University, AL, USA

**Keywords:** Bone Mineral Density, Osteoporosis, Asian-Indians, Predictors, Risk Factors

## Abstract

**Background:** Osteoporosis is a systemic skeletal disorder characterized by low bone mineral density (BMD) that leads to an increase in bone fragility, causing an individual to be at an increased risk for fractures. Asian-Indians are at an increased risk for developing osteoporosis. Considering the number of Asian-Indians in the US is rapidly growing, they likely could be an underappreciated population at risk for bone fractures. The aim of this study was to investigate bone health and determine the factors affecting BMD in Asian-Indians living in the US.

**Methods:** Asian-Indians residing in Northern Mississippi (*n* = 87) were enrolled in this cross-sectional study from June 2013 to August 2014. Eligible participants completed a self-administered Osteoporosis Risk Factor Assessment questionnaire. BMD and body composition were measured using a dual energy x-ray absorptiometry scan (DXA).

**Results:** Eight-seven Asian-Indians (male: 62.1%) participated, with the average age being 28.49 yr old (SD = ±6.62). Overall, 31.0% and 48.3% had low femoral neck BMD and spinal BMD, respectively. Multiple regression analysis revealed that age, percent body fat, and body mass index (BMI) significantly predicted BMD at femur neck (*P*<0.05). Additionally, percent body fat, BMI, childhood milk consumption, and gender were statistically significant predictors of spinal BMD (*P*<0.05).

**Conclusions:** The findings from this study should be beneficial to healthcare providers that work with Asian-Indian population groups. Health promotion programs focusing on osteoporosis prevention are needed among Asian-Indians to prevent the risk of fractures.

## Introduction

Osteoporosis is a disease that weakens bone tissue resulting in an increased risk of bone fracture^1^. Based on estimation of the National Health and Nutrition Examination Survey 2005-2010; 10.2 million older adults in United States in 2010 had osteoporosis and 43.4 million older adults had low bone mass^2^. Measuring a person’s bone mineral density (BMD) allows osteoporosis to be identified^3^. If the BMD is -2.5 standard deviations (SDs) or more below “normal” bone density quantity, then it is diagnosed as osteoporosis^3^. Osteopenia, often referred to as the precursor to osteoporosis, occurs when a person’s BMD is between -1 to -2.5 SDs below “normal” bone density quality^4^.

Many factors contribute to the development of low BMD and osteoporosis. Several lifestyle factors can be modified to prevent the reduction of bone from occurring, while other factors cannot be changed^5^. Calcium intake is an important variable that greatly contributes to bone health^6^. Bones absorb calcium in order to become both stronger and larger. Consuming an adequate amount of calcium promotes bone formation and maintains suitable bone health^7^. Exercise also promotes bone growth. Participating in regular weight bearing physical activity, especially at a young age, increases the buildup of bone mass^8^. Sedentary lifestyle is an important risk factor for developing osteoporosis. Inactivity allows bone reabsorption to surpass bone formation, which can lower the bone density^9^. Alcohol consumption and cigarette smoking reduce the absorption of calcium and increase osteoclast activity increasing the risk of developing low BMD and osteoporosis^10,11^.

Age, gender, and family history are non-modifiable factors that contribute to low BMD. Bone density is known to decrease with age^8^. Childhood and adolescence are pivotal years for developing peak bone density. During this time, bones are better able to increase their density compared to later ages^12^. Bone density reaches its peak around the third decade of life and steadily decreases throughout adulthood^13^. Women are more likely to develop low BMD or osteoporosis than men^14^. A family history of osteoporosis and low bone density increases the risk of developing the disease, especially for postmenopausal women^15^.

In 2010, there were 2,765,155 Asian-Indian immigrants in the United States, making them the second largest immigrant group^16^. Both osteoporosis and low BMD are highly prevalent among Asian-Indian men^17^. In 2008, an estimated 25 million Asian-Indians worldwide were predicted to have osteoporosis, with the number expected to rise each year^17^. This population typically has a lower BMD than Caucasians^18^. Osteoporotic fractures occur 10 to 20 yr earlier in Asian-Indian men and women when compared to their Western Caucasian counterparts^19^.

The aim of this study was to investigate the bone health and determine the predictors of BMD in the Asian Indian community residing in United States. The findings from this study will be beneficial to healthcare providers or public health professionals for developing osteoporosis prevention and management interventions particularly targeted for the Asian Indian population group.

## Methods

### Participants and Procedures

Participants were recruited from June 2013 to August 2014, utilizing two different techniques, convenience sampling and snowball sampling. Study recruitment included posting flyers and mass emails sent to potential participants at a large Southern University. Once participants began contributing to the study, the snowball sampling technique was utilized by having current participants inform other potential participants about the study.

Potential participants were called for a brief phone interview for study eligibility. Participants had to be at least 18 yr old to participate. They were asked three, yes-or-no questions, to which they had to answer “yes” to at least one of the three questions to qualify for participation in the study. The three questions asked were as follows: 1) “Were you born in India?” 2) “Were both of your parents were born in India?” and 3) “Were all of your grandparents were born in India?” The female participants were asked if they were currently breastfeeding, to which they had to answer “no” to gain entry into the study. Eligibility was also denied if they were on medications or had certain diseases known to increase one’s risk for osteoporosis.

### Measures

If judged eligible after the phone interview, participants were emailed questionnaires to complete regarding their bone health. If participants did not have Internet access, they were instructed to pick up and return the questionnaires in the Bone Density Laboratory at (The University of Mississippi). The questionnaires assessed the participants’ weekly calcium intake, family history of osteoporosis, alcohol and smoking habits, age, gender, physical activity involvement, and menstrual status for females.

After completing the questionnaires, participants then scheduled a time to have a DXA scan. During the DXA scan visit, height and weight were measured using the standard doctor’s scale. Body composition, lumbar spine, femoral neck BMD, was measured using a Hologic Delphi-W (Bedford, MA) dual energy x-ray absorptiometry machine.

### Statistical Analysis

Descriptive statistical analyses were performed to describe and summarize the data. For inferential statistics a standard multiple linear regression was performed. Results were statistically significant if *P*-value was less than or equal to 0.05.

### Ethical Considerations

Consent was obtained from participants before they were allowed into the study. Participants were informed on potential risks the study presented and the importance of the study. This study was performed with the approval of the University’s Institutional Review Board.

## Results

Eighty-seven participants took part in this study. Fifty-four were males with the remaining 33 being female. All participants were of Asian-Indian decent, with ages ranging from 18 to 49 yr, with the average age being 28.49 yr old (SD=6.62). Average time for living in the United States was 31.78 (SD=8.34) yr. Characteristics of study participants are presented in Table 1.

**Table 1:** Characteristics of study participants

**Variables**	**Number**	**Percent**
Childhood milk consumption		
None	4	4.7
1 serving/day	22	25.6
2 servings/day	44	51.2
3 servings/day	14	16.3
4 or more servings/day	1	1.2
5 servings/day	1	1.2
High school sports participation		
No	22	25.3
Yes	65	74.7
Gender		
Male	54	62.1
Female	33	37.9
Smoking		
No	80	93.0
Yes	6	7.0
Consuming alcohol		
No	30	34.5
Yes	57	65.5
Dairy intake		
Inadequate	36	41.4
Adequate	51	58.6
Body mass index		
Normal	43	49.4
Overweight	36	41.4
Obese	8	9.2
Family history of osteoporosis		
No	70	80.5
Yes	17	19.5
Total Spine		
Normal	45	51.7
Low (osteopenia/osteoporosis)	42	48.3
Femur Neck		
Normal	60	69.0
Low (osteopenia/osteoporosis)	27	31.0
Total Femur		
Normal	81	93.1
Low (osteopenia/osteoporosis)	6	6.9

Regression analysis revealed age (*β*=-0.257, *P*=0.02), total body fat percentage (*β*=-0.576, *P*=0.006), and BMI (*β*=0.464, *P*=0.001) were significant predictors of BMD in the femoral neck. The findings of relationship between variables and femur neck are presented in Table 2.

**Table 2:** Summary of regression analysis of variables related to femur neck (dependent variable)

**Variables**	**Unstandardized Coefficient (B)**	**SE**_B_	**Standardized coefficient (β)**	***P *** **value**
Age (yr)	-0.036	0.015	-0.257	0.020
Total body fat %	-0.066	0.023	-0.576	0.006
Childhood milk consumption	0.231	0.119	0.213	0.055
High school sport participation	0.025	0.230	0.011	0.914
Gender	0.655	0.391	0.340	0.098
Smoking	0.215	0.385	0.059	0.578
Dairy intake	-0.098	0.080	-0.140	0.227
Family history of osteoporosis	-0.096	0.255	-0.041	0.707
Body mass index	0.128	0.037	0.464	0.001
Alcohol consumption	-0.419	0.225	-0.212	0.067

F (10, 74) = 2.691, p = 0.007, R2 (Adjusted R2) = 0.267 (0.168)

Body fat percentage (*β*=-0.482, *P*=0.029) and BMI (*β*=0.443, *P*=0.003) were significant predictors of total femur BMD. Table 3 presents relationship between variables and total femur.

**Table 3:** Summary of regression analysis of variables related to total femur (dependent variable)

**Variables**	**Unstandardized Coefficient (B)**	**SE**_B_	**Standardized coefficient (β)**	***P *** **value**
Age (yr)	-0.017	0.015	-0.13	0.256
Total body fat %	-0.051	0.023	-0.482	0.029
Childhood milk consumption	0.037	0.116	0.037	0.751
High school sport participation	-0.059	0.224	-0.030	0.791
Gender	0.736	0.382	0.415	0.058
Smoking	0.190	0.376	0.056	0.614
Dairy intake	-0.120	0.078	-0.187	0.129
Family history of osteoporosis	-0.022	0.249	-0.010	0.928
Body mass index	0.112	0.036	0.443	0.003
Alcohol consumption	-0.325	0.220	-0.178	0.144

F(10, 74) = 1.588, p = 0.127, R2 (Adjusted R2) = 0.177 (0.065)

Furthermore, total body fat percentage (*β*=-0.451, *P*=0.038), childhood milk consumption (*β*=0.286, *P*=0.014), gender (*β*=0.529, *P*=0.014), and BMI (*β*=0.382, *P*=0.009) are significant predictors of total spine. The results of relationship between variables and total are presented in Table 4.

**Table 4:** Summary of regression analysis of variables related to total spine (dependent variable)

**Variables**	**Unstandardized Coefficient (B)**	**SE**_B_	**Standardized coefficient (β)**	***P *** **value**
Age (yr)	-0.018	0.017	-0.120	0.291
Total body fat %	-0.055	0.026	-0.451	0.038
Childhood milk consumption	0.330	0.132	0.286	0.014
High school sport participation	0.265	0.255	0.115	0.301
Gender	1.087	0.434	0.529	0.014
Smoking	0.106	0.427	0.027	0.805
Dairy intake	-0.127	0.089	-0.170	0.160
Family history of osteoporosis	0.233	0.284	0.093	0.415
Body mass index	0.112	0.042	0.382	0.009
Alcohol consumption	-0.445	0.250	-0.211	0.079

F(10, 73) = 1.967, p = 0.050, R2 (Adjusted R2) = 0.212 (0.104)

## Discussion

We found that the males in this study had lower BMD than females. These findings on gender and BMD were not in line with other studies^1^. Women have been shown to experience low bone density and osteoporosis at an earlier age than men due to men’s higher peak bone mass^14^, making this finding quiet unique. This could be because our sample did not have many postmenopausal women and the women constituting our sample were young. Menopause severely affects the female’s BMD, so with a large group of younger females, their bone density would be higher than older women’s would be. In addition, a small sample without adequate power for subgroup analysis could have contributed to the non-significant results. Further investigation is needed in this regard.

Age and BMD had an inverse relationship. Studies have shown that while age increases, bone density decreases^3,8^. Our study showed that this negative correlation was only evident in the femoral neck. Total femur and spine were not shown to have any significant decrease as the age of the participants increased. This may also be related to relatively small sample size of our study. For future studies, widely advertising the study in nearby cities and allowing the snowball sampling to continue from there might elicit greater participation.

Both percent body fat and BMI had significant effects on the BMD at all three measured sites. Percent body fat and BMD displayed an inverse relationship, while a direct relationship was found between BMI and BMD. Fat mass has been established as a factor that influences bone density^20^. When there is a greater amount of fat-mass compared to lean-mass, BMD declines. Lean-mass, or fat-free mass, puts an added strain on the bones. This increases bone density during physical activity when the bone is being strained and has forces being applied to it. Low BMI and low body weight are risk factors for developing low BMD^21^, which supports our findings that those with a lower BMI had a lower bone density.

BMD in total spine were significantly affected by the amount of milk the participants consumed during childhood. Frequent consumption of milk during childhood and adolescence elicited increases in BMD later in life. Taking in an adequate amount of calcium during these pivotal years can allow one to attain a higher peak bone mass, creating stronger bones^6^. We did not find the femur notably affected by childhood milk intake. This could be due to cross-sectional design of this study. In addition, since the study was limited to cross-sectional design, it cannot be confirmed that the results found on this population mirror the same results other Asian-Indians in the United State may display.

This study found that alcohol consumption had an effect on the BMD of the femoral neck and total spine that was approaching significance. Alcohol and bone density had an inverse relationship with each other, displaying that as alcohol consumption increased, BMD decreased. Consuming heavy amounts of alcohol is detrimental to bone health. However, consuming moderate amounts of alcohol, about two glasses per day, is associated with an increase in BMD^22^. Further investigations are needed to discern alcohol’s effect on bone. Caution must be exercised when interpreting results because responses, since this study used self-reported questionnaire. Self-report biases allow participants to obscure the truth by exaggerating or under-reporting the truth. Their responses also rely on recalling certain moments from their past, which can be unintentionally misremembered.

## Conclusions

Based on the findings of this study, percent body fat and body mass index predicted both femur neck and spinal BMD. With the data gained from this study, it is evident that Asian-Indians are at a risk for developing osteoporosis and low BMD. The findings from this study should be beneficial to healthcare providers that work with Asian-Indian population groups. Health promotion programs focusing on osteoporosis prevention are needed among Asian-Indians to prevent the risk of fractures.

## Acknowledgments

The authors would like to thank all the participants who participated in this research study.

## Conflict of interest statement

None.

## Highlights

- Thirty-one percent had low femoral neck bone mineral density (BMD).
- Forth-eight percent had low spinal BMD .
- Percent body fat and body mass index predicted both femur neck and spinal BMD.
